# How and When Generalized Reciprocity and Negative Reciprocity Influence Employees’ Well-Being: The Moderating Role of Strength Use and the Mediating Roles of Intrinsic Motivation and Organizational Obstruction

**DOI:** 10.3390/bs13060465

**Published:** 2023-06-03

**Authors:** Nan Zhu, Yuxin Liu, Jianwei Zhang

**Affiliations:** 1 School of Business Administration, Fujian Jiangxia University, No.2 Xiyuangong Road, University Town, Fuzhou 350108, China; 2 Business School, University of International Business and Economics, Rm. 822, Keyan Building, No.10, Huixin Dongjie, Beijing 100029, China; 3 School of Humanities and Social Science, Beijing Institute of Technology, Rm. 411, Zhongxin Teaching Building, 5 South Zhongguancun Street, Beijing 100081, China

**Keywords:** generalized reciprocity, negative reciprocity, well-being, intrinsic motivation, perceived organizational obstruction

## Abstract

Although the literature has shown that generalized reciprocity and negative reciprocity as exchange norms can significantly influence employees’ outcomes, knowledge about how and when the two types of norms influence employees’ well-being is limited. Based on social exchange theory and self-determination theory, we built and investigated a model by conducting a large questionnaire survey with 551 employees and managers. The results of the structural equation model supported our hypotheses. First, generalized reciprocity is positively related to well-being, and negative reciprocity is negatively related to well-being. Both intrinsic motivation and perceived organizational obstruction can meditate roles in the above relationships. Moreover, strength use can enhance the relationship between generalized reciprocity and intrinsic motivation, and it can also weaken the relationship between negative reciprocity and perceived organizational obstruction. Our research represents a significant step towards better understanding the work-related implications of imbalanced reciprocity, highlighting the destructive influence of negative reciprocity on employees’ well-being.

## 1. Introduction

An organization expects to build a balanced exchange norm (balanced reciprocity, i.e., BR: the organization offers help to employees in exchange for a quick and equal return) to affect employees’ rewards for it [1,2,3,4]. However, it is difficult for employees to offer an equal return immediately, thus decreasing the organization’s willingness to maintain the norm for a long period. In contrast, two types of imbalanced reciprocity [1,2], generalized reciprocity (GR: the organization provides employees benefits without asking for quick return) and negative reciprocity (NR: the organization realizes a self-serving goal while hurting employees’ benefits), can stably exist in the workplace. This is because they can significantly impact employees’ rewards to the organization (e.g., helping behavior, positive interpersonal relationships, deviant behaviors, executive function, and task performance) [5,6,7,8,9]. However, most of the literature based on social exchange theory (SET) [1] mainly focuses on employees’ extrinsic rewards to the organization (e.g., productivity) [10], lacking research on intrinsic rewards (e.g., well-being) [1,11]. Extrinsic benefit exchange is only a means to win intrinsic benefits [1]. Employees’ well-being in the organization can be viewed as an intrinsic reward because it reflects their affection and recognition for the organization. How and when do GR and NR influence employees’ well-being? Intrinsic rewards (e.g., well-being) can serve as inducements to continue to supply benefits, and the integrative bonds created in the process fortify the social relationship [1]. Therefore, it is critical to explore how and when GR and NR influence employees’ well-being.

Employees’ well-being involves not only employees’ perceptions about their work and life satisfaction but also their psychological experience and the level of satisfaction exhibited in both their work and personal lives, so it comprises three dimensions: workplace well-being, psychological well-being, and life well-being [12]. To date, the majority of the literature has shown that individuals’ well-being has a positive effect on their outcomes, such as health [13], high-quality interpersonal relationships [14], loyalty [15], and green behavior [16].

Although the literature has demonstrated that BR can increase well-being [17], the influences of GR and NR on employees’ well-being have been hitherto unexplored. Gaining a better understanding of the impact of imbalanced reciprocity on well-being is significant for the following reasons. First, exploring the effects of GR and NR on well-being could help researchers better understand how well-being, as employees’ intrinsic reward to the organization, is affected by exchange norms. The research on employees’ required contribution to the organization mainly focuses on extrinsic rewards, ignoring intrinsic rewards [1,11]. Researching intrinsic rewards (e.g., well-being) is significant for the organization because it tends to increase employees’ willingness to continue their association with the organization due to the personal affection and recognition of the organization. A person’s attraction to a group stimulates his desire to become/keep a member [1]. The more attractive the individual is to an organization, the more cohesive the group will be. Additionally, our research could help practitioners improve employees’ well-being by improving current imbalanced reciprocity.

**Figure 1:**
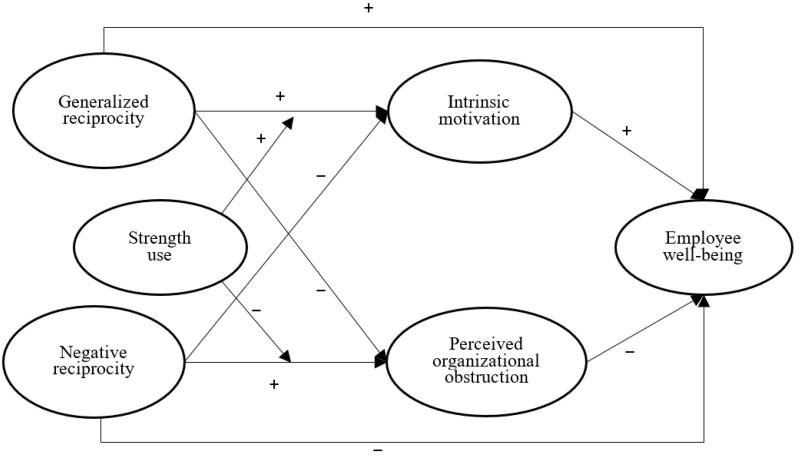
The theoretical framework.

The purpose of this paper is to explore the influences of GR and NR on employees’ well-being. Moreover, intrinsic motivation (IM) and perceived organizational obstruction (POB) play mediating roles in the relationship between imbalanced reciprocity and well-being. Then, we propose that employees’ strength use tends to enhance the increasing effect of GR on IM and alleviate the increasing effect of NR on POB. Specifically, we build a theoretical framework (Figure 1) by drawing upon social exchange theory (SET) and self-determination theory (SDT). First, an organization with GR tends to increase employees’ well-being. Remaining obligated to others as well as trusting them to discharge their obligations for considerable periods can fortify social bonds [1]. The organization with GR retains this obligation to employees to provide unrequited benefits for them because the organization trusts that employees can give them a long-term return. An organization’s trustworthiness may increase employees’ trust in the organization to increase employees’ well-being [18]. In contrast, an organization with NR can significantly decrease employees’ organizational trust [18] to decrease well-being [19]. Second, the degree of employees’ intrinsic motivation can be augmented by an increase in the perception of their authentic interests [20]. Therefore, GR and NR tend to increase or decrease employees’ perceptions of their authentic interests to influence intrinsic motivation. Meanwhile, POB is also likely to mediate the relationship between reciprocity and well-being. SET suggests that social norms reflect a subordinate’s evaluations of a superior’s demands [1,21]. NR usually harms employees’ interests [1,4] to increase their negative evaluations of the organization (e.g., perception of the organizational obstruction). Moreover, GR increases employees’ perception of real interests to decrease POB. Third, SDT assumes that individuals’ behaviors can satisfy basic psychological needs to facilitate their natural growth [22]. Employees may increase the trust of an organization using personal strength because it helps to increase work efficiency. In the GR organization, the employees who usually use their strength will increase the organization’s trust to strengthen the increased intrinsic motivation. Employees who use their strengths may experience less POB in NR organizations.

Our research has specific theoretical and practical implications. First, based on SET, our research expands the literature on intrinsic rewards of employees to the organization by investigating the effects of GR and NR on employees’ well-being. Our research proves that an organization’s expectation relative to exchange relationships with employees can impact the intrinsic rewards of employees to the organization. The existing research on the required contributions of employees to the organization mainly focuses on extrinsic rewards (e.g., task performance), ignoring intrinsic rewards [1,11]. However, intrinsic rewards reflect employees’ affection for the organization, which can boost the long-term relationship between employees and the organization. Second, we explored the antecedents of employees’ well-being from the imbalanced reciprocity perspective, expanding the literature on well-being. Third, based on SET [1], we bridged the unclear links between imbalanced reciprocity and well-being by uncovering the mediating roles of IM and POB, thus helping researchers better understand the psychological mechanism of social exchange. Fourth, by integrating SET [1,4,18] and SDT [22], our research sheds light on how employees actively enhance or alleviate the impact of GR and NR on their psychological states and motivation by examining that the efficacy of GR and NR is contingent on employees’ strength use. Overall, our research complements prior SET- and SDT-based well-being studies from the imbalanced reciprocity view.

## 2. Theoretical Background and Hypotheses

### 2.1. The Intrinsic and Extrinsic Rewards Perspective of SET on Reciprocity

The organization provides employees with material and social rewards in exchange for their loyalty and effort [23,24]. More importantly, the literature based on SET suggests that employees can also provide rewards to their organization in return, and the rewards are mainly divided into intrinsic and extrinsic categories [1,11,25]. Intrinsic rewards mainly reflect that the employees are attracted by the organization and expect to be accepted by the organization as well as accept the organization psychologically (e.g., affection, attraction, recognition, love, respect). Extrinsic rewards reflect that employees approve organizational requirements and provide instrumental services to the organization (e.g., approval of decisions or opinions, instrumental services, money, and physical labor) [1,11,25]. The distinction between intrinsically and extrinsically rewarding social associations is that extrinsic reward associations can be viewed as a means to further benefits, while intrinsic reward associations do not involve these benefits [1]. “Extrinsic benefits constitute standards for comparing associations and deciding between them, whereas no such independent criteria of comparison exist for intrinsically rewarding associations.” [1] (P58). This distinction means that employees who provide extrinsic rewards to the organization may compare the benefits provided by different organizations. Employees may then give up the connection with the current organization because they tend to accept the higher benefits from other organizations, thus increasing the difficulty of maintaining a long-term social relationship with the organization. The literature on SET mainly focuses on extrinsic rewards of employees to the organization and lacks research on intrinsic rewards in the case of social exchanges [11]. SET suggests that the “exchange of extrinsic benefit is merely meant for expressing and winning intrinsic benefits” [1].

Regarding the outcomes of GR and NR, thus far, the current literature mainly studies their influences on employees’ work attitudes and outcomes from the perspectives of intrinsic and extrinsic rewards [3,18,26,27,28]. For example, from a extrinsic reward viewpoint, GR can decrease turnover intention [3,27] and increase training participation, cooperative behaviors, and task performance [9,29,30]. In contrast, NR can prompt employees’ deviant behaviors and turnover intention and decrease task performance [5,9,18]. Furthermore, most of the literature focuses on the influences of GR and NR on employees’ outcomes from an extrinsic reward viewpoint. However, there is limited research on work outcomes from the intrinsic rewards viewpoint. For example, from the intrinsic rewards viewpoint, GR can increase affective commitment and psychological empowerment [3,18,26,27,28], job satisfaction, and organizational trust [31] and can attenuate psychological empowerment, organizational trust [18], and affective commitment [3].

Employees’ well-being can be seen as employees’ intrinsic reward to the organization. To date, empirical research has demonstrated that BR can significantly increase individuals’ well-being [17]. However, whether and how GR and NR influence employees’ well-being differently has not yet been explored. Based on SET [1], well-being can be viewed as an intrinsic reward to the organization because employees high in well-being are attracted to the organization. This intrinsic attraction tends to increase their willingness to continue social exchange relationships with the organization [1] and maintain a long relationship with the organization. The greater the attraction of employees to an organization, the more cohesive the organization is [1]. Meanwhile, employees’ improvement in well-being indicates that they accept organizational social exchange norms. Employees also expect to obtain social acceptance from the organization, thus providing intrinsic rewards to the organization. Therefore, exploring the impacts of GR and NR on employees’ well-being could help researchers better understand how to maintain the cohesion of organizations from an intrinsic reward perspective. The exploration of this issue can also help practitioners expand the ways for organizations to maintain long-term social relationships with employees.

### 2.2. The Effects of Generalized Reciprocity and Negative Reciprocity on Employees’ Well-Being

Employees’ well-being involves not only employees’ perceptions and feelings about their work and life satisfaction but also their psychological experience and the level of satisfaction exhibited in both their work and personal lives, and thus, it comprises three dimensions: workplace well-being, psychological well-being, and life well-being [12]. Well-being can be viewed as employees’ intrinsic reward for the organization because it reflects that the employees are attracted to their organization and the attachment to their organization [1]. SET focuses on the formation of social exchange relationships [1]. It assumes that reciprocated benefactions can create social bonds among different parts [1]. The benefits each supply to the others can be seen as rewards, which create integrative bonds to enhance social relationships [1]. Employees’ well-being as intrinsic rewards tends to strengthen the social relationship with the organization. Well-being is regarded as both the various life (e.g., satisfaction with family and social life) and work satisfaction (e.g., satisfaction with the job itself) [14]. The literature summarizes that the antecedents of employees’ well-being include organizational context, traits, the psychological states [14,15,32,33,34,35,36,37,38], and work behavior [39,40], such as autonomy support [41,42] and challenge and hindrance demands [43,44]. BR usually forms a relationship context in the workplace, which can increase individuals’ well-being [17]. However, can imbalanced reciprocity (i.e., GR, NR) enhance or decrease employees’ well-being? This issue has not been explored so far. Exploring this issue stands to help the researcher better understand whether imbalanced reciprocity can influence employees’ intrinsic rewards for the organization to forty social relationships.

GR and NR tend to facilitate or restrain employees’ well-being because of the difference in organizational trust they perceive during the work. When both parties abide by the exchange rules, they tend to build a more trusting and loyal relationship [2]. First, since social exchange requires trusting others to reciprocate, the preliminary problem is to prove that you are trustworthy [1]. The organization with GR expects to maintain long-term social exchange relationships with employees. The organization regularly performs an altruistic duty to prove itself worthy of further trust [1]. That is why an organization with GR generously offers benefits to employees without stipulating quick returns [1,18]. One key issue for understanding the reciprocity of SET is how employees evaluate the kindness of an action [45]. Employees working in the GR organization are likely to experience organizational hospitality, kindness, and altruism [4,46]. Next, employees will feel a high level of organizational trust [18], which in turn enhances their psychological well-being [19].

In contrast, organizations with NR do not trust employees to achieve long-term exchange relationships. That is why an organization with NR can sacrifice employees’ interests to realize the interests of the organization [4,18]. SET assumes that ingroup pressures are rooted in the process of social exchange [1]. Employees working in the NR context are likely to experience inner pressure to decrease organizational trust [18], which in turn impairs psychological well-being [19]. Therefore, we propose the following:

**Hypothesis** **1a.** *GR is positively related to well-being.*

**Hypothesis** **1b.** *NR is negatively related to well-being.*

### 2.3. The Mediating Role of Intrinsic Motivation

Intrinsic motivation refers to the desire to expend effort based on interest in and enjoyment of the work itself [20,33]. The current literature on SET suggests that the organizational context can encourage employees to give back or reward their organization through intrinsic motivation [20]. To date, researchers have increased their focus on the mediating role of IM in the associations between contextual factors and employees’ well-being [47,48]. For example, work hope goals can increase individuals’ motivation, which in turn influences their well-being [49], and job demands significantly influence employees’ well-being through autonomous motivation [44]. IM is one important facet of autonomous motivation [44]. It has been demonstrated to be positively related to employees’ well-being [48]. A basic tenet of SET is that relationships evolve into trusting, loyal, and mutual commitments as long as the parties abide by certain “rules” of exchange [2]. We propose that GR and NR may increase or decrease employees’ well-being through IM. It stands to help managers and researchers understand how GR and NR exert different effects on employees’ well-being in the workplace to prompt the build-up of employees’ psychological mechanisms.

According to SET, an organization with GR context tends to provide altruistic help for employees without the requirement of return [4,18], because this altruistic reciprocity focuses on long-term repayment [50,51]. Organizational altruistic help tends to realize employees’ interests. Based on SET, Mitchell et al. [52] suggested that personal interest is a major guiding motivational force of interpersonal exchange. The degree of intrinsic motivation of workers increases with an increase in the perception of their authentic interests [20]. So, GR is likely to increase employees’ intrinsic motivation. Meanwhile, an organization with GR tends to attain employees’ interest, which may promote their trust in the organization. The current literature has demonstrated that GR characterized by providing material rewards or help for employees is significantly positively related to employees’ organizational trust [18]. This trust helps employees easily perceive meaningful information provided by the organization for self-competence to increase employees’ IM. For example, monetary rewards can increase intrinsic interest when pay provides meaningful information regarding self-competence in the workplace [53]. Second, the current literature on SET suggests that intrinsically motivated employees feel an obligation to reciprocate [20,54], such as by providing intrinsic rewards for their organization (e.g., well-being). Previous studies confirm that IM is positively related to employees’ well-being [48]. Therefore, IM may mediate the relationship between GR and IM.

By contrast, an organization with NR requires employees to return benefits immediately [4,18], so it focuses on short-term returns. An organization with NR tends to harm employees’ interests to realize organizational interest [4,18], thus increasing the difficulty for employees to realize personal interest to decrease their organizational trust. Current literature has demonstrated that NR is negatively related to trust [18]. Then, employees have difficulty perceiving meaningful information from the organization to decrease IM [53]. So, IM is likely to mediate the relationship between NR and well-being. We propose that:

**Hypothesis** **2a.** *IM mediates the relationship between GR and well-being.*

**Hypothesis** **2b.** *IM mediates the relationship between NR and well-being.*

### 2.4. The Mediating Role of Perceived Organizational Obstruction

Based on SET, POB is regarded as “an employee’s belief that the organization obstructs, hinders or interferes with the accomplishment of his or her goals and is a detriment to his or her well-being” ([24], P667). It reflects employees’ evaluation of the negative or harmful nature of their relationship with their organization [55]. When employees perceive negative treatment and harm from the organization, they tend to see the organization as a source of negative treatment, whereas little research has explored the organization as a source of negative treatment [24]. The negative nature of POB can significantly connect the perception of an imbalanced relationship and a positive psychological state [56]. POB can be seen as a suitable mediator that can explain the indirect relationships between imbalanced reciprocity (i.e., GR, NR) and well-being. Exploring POB mediating effects helps researchers better understand the psychological process of the effects of GR and NR on employees’ intrinsic rewards for the organization.

An organization with NR attempts to achieve benefit at the expense of hurting employees’ interests [4]. NR tends to make the work process difficult, thus leaving employees to feel that work goals are difficult to achieve in order to perceive POB. Gibney et al. [55] suggest that when employees believe that the organizations’ treatment makes work processes more difficult, the obstruction will be perceived. So, employees are apt to perceive a high level of POB when they work in an NR organization. For example, the perception of negative treatment in the workplace causes increased POB [56]. Furthermore, POB is described as a detriment to the organization to employees’ well-being [24]. It predicts the cognitive separation in individual and organizational identities [55]. Specifically, when employees perceive a hinderance to goal accomplishment from the organization, they will feel conflicted, embarrassed, or even ashamed about being part of this organization. Then, they will experience an unpleasant psychological state that decreases their well-being.

In contrast, organizations with GR tend to provide altruistic help to employees [4,18], thus letting employees more easily perceive that the work procedure is simple to conduct to decrease POB. Then, employees are likely to feel a pleasant psychological state to increase their well-being. So, we propose that:

**Hypothesis** **3a.** *POB mediates the relationship between NR and well-being.*

**Hypothesis** **3b.** *POB mediates the relationship between GR and well-being.*

### 2.5. The Moderating Role of Strength Use

Strength use means that individuals use their most prominent advantages more frequently or in new ways [57,58]. Employees’ strength can be a distinctive characteristic or a capacity that motivates employees to achieve their optimal function and development [59], including physical, psychological, or character strength (e.g., intelligence, knowledge, skills, competence) [60]. Employees can utilize their strengths to maximize the positive benefits [60,61]. Strength use tends to be intrinsically motivating and allows employees to achieve the best functionality [57,62], such as in self-esteem, self-efficacy, and vitality [63,64,65]. The limited literature has hitherto examined the moderating role of strength use on the relationship between context and employees’ psychological state. We propose that strength use enhances the positive effect of GR on IM and buffers the negative effect of NR on POB. This exploration stands to further improve the inner state of employees under the beneficial or adverse conditions of positive psychology.

First, strength use tends to enhance the trust of the organization that implements GR to the employees, thereby increasing the trust of the employees in the organization, and finally enhancing the increasing trend of employees’ IM. SET proposes that social exchange promotes and depends on trust [1]. Why GR can improve employees’ trust in the organization [18] mainly depends on the organization’s trust in employees. Strength use can increase the trust of an organization with GR to its employees. SDT proposes that individuals’ behaviors can satisfy basic psychological needs to facilitate their natural growth [22]. Strength use can significantly satisfy individuals’ basic needs [23] to increase work meaningfulness and efficiency [63]. Then, an organization with GR will enhance its trust in employees. Then, strength use is likely to facilitate the trust of the organization with GR to employees. SET proposes that social exchange promotes trust [1]. When employees perceive the organization’s trust in them, they will have more trust in the organization [18]. Then, employees may enhance their skill development and invest their interests in their work, thereby promoting the upwards trend of intrinsic motivation in the GR organization.

Second, strength use may weaken the NR organization’s distrust of employees to reduce employees’ distrust of the organization, and finally weaken the upwards trend of POB of employees. NR reduces employees’ trust in the organization [18] mainly because the organization with NR does not trust employees. Strength use will decrease NR organizations’ distrust of employees. Specifically, strength use can significantly satisfy individuals’ basic needs [66] to increase work meaningfulness and efficiency [63]. Then, strength use can help the employees who work in an NR organization reduce their distrust of employees. In particular, an organization with NR can perceive that employees use their strength to achieve work goals, which can bring short-term benefits to trust employees. Employees’ levels of POB will vary following their interpretations of the treatment they receive from the organization [55]. Strength use tends to reduce employees’ distrust of the NR organization to weaken the increasing trend of POB. So, employees can feel less POB by using their strengths in the NR organization. Accordingly, we propose that:

**Hypothesis** **4a.** *The positive relationship between GR and IM becomes strong when strength use is high rather than low.*

**Hypothesis** **4b.** *The positive relationship between NR and POB becomes weak when strength use is high rather than low.*

## 3. Method

### 3.1. Participants and Procedure

This study conducted a large-scale questionnaire-based survey in China using a cross-sectional design. The sample comprised 727 employees and managers (randomly selected) who mainly worked in one information technology corporation, petroleum processing plant, biotechnology corporation, and manufacturing corporation, respectively. Initially, four volunteers from the Executive Development Program (EDP) of the university in China communicated with the human resource managers or the CEOs of corresponding corporations and explained our research aim and content to them. They also explained that we would provide free occupational training courses for those employees and managers who were willing to participate in the questionnaire survey. Finally, the above four corporations agreed to take this survey. We visited these corporations and informed participants that the survey would be anonymous, and that personal information would be confidential before the survey. We also informed them that the results of the survey are only used for academic research. Before the survey (time 1), we explained to the participants that the questionnaire was divided into two parts, and only the first part of the questionnaire would be distributed this time, and the second part of the questionnaire would be distributed one week later (time 2). At time 1, the first part of the questionnaire (i.e., the measurement items of GR, NR, and strength use) was distributed to participants. After 1 week, at time 2, the second part of the questionnaire (i.e., the measurement items of IM, POB, well-being, and demographic information) was distributed to participants (all the measurement items are shown in the Appendix A).

At last, we required participants to bind the two parts of the questionnaire together and return them to us at the end of the training. After deleting the questionnaires with missing values, the final valid sample size of 551 was studied for the present research, with an response rate of 75.8%. The mean age of the participants was 30.6 years (SD = 5.7), And 62.8% were male. The average length of service in the current position is 3.66 years. Most of them held a university degree (junior college, 19.6%; undergraduate, 72.1%; postgraduate, 6.5%; others, 1.8%). Participants covered different kinds of positions (administrator, 12.3%; production, 31.6%; marketing and sales, 17.8%; supporting service, 11.1%; accountancy and finance, 5.8%; technical research and development, 17.6%; other, 3.8%).

### 3.2. Measures

#### 3.2.1. Generalized Reciprocity and Negative Reciprocity

Wu et al. [18] designed a reciprocity scale including four items for GR, and seven items for NR (e.g., “My organization would help me develop myself, even if I cannot make more contributions at present” and “My organization expects more from me than it gives me in return”). A seven-point Likert scale was used to assess all items ranging from “7 = totally agree” to “1 = totally disagree”. The Cronbach’s alpha for GR and NR was 0.80, and 0.91, respectively.

#### 3.2.2. Intrinsic Motivation

Gagné et al. [67] developed a scale including three items for intrinsic motivation (e.g., “because the work I do is interesting.”). A seven-point Likert scale was used to assess all items ranging from “7 = totally agree” to “1 = totally disagree”. Cronbach’s alpha was 0.80.

#### 3.2.3. Perceived Organizational Obstruction

Gibney et al. [24] developed a POB scale including five items (e.g., “the company blocks my personal goals”). A seven-point Likert scale was used to assess all items ranging from “7 = totally agree” to “1 = totally disagree”. Cronbach’s alpha was 0.93.

#### 3.2.4. Strength Use

Huber et al. [60] developed the strength use scales including fourteen items (e.g., “I use my strengths every day.”). We used eleven items to measure strength use because three items have low values of factor loadings. A seven-point Likert scale was used to assess all items ranging from “7 = totally agree” to “1 = totally disagree”. Cronbach’s alpha was 0.94.

#### 3.2.5. Well-Being

Zheng et al. [12] developed an employee well-being scale with eighteen items for life well-being, workplace well-being, and psychological well-being (e.g., “I find real enjoyment in my work.”). A seven-point Likert scale was used to assess all items ranging from “7 = totally agree” to “1 = totally disagree”. Cronbach’s alpha was 0.92.

#### 3.2.6. Control Variables

We took age, gender, and tenure variables into the examination of the whole model to rule out the possibility that these control variables influence our expected relationships. For example, the level of men’s well-being declined as they grew older, and working years are also significantly associated with well-being (e.g., satisfaction with work) [68]. The results of descriptive statistics and structural equation model (SEM) indicated that the above control variables were not significantly related to all the constructs including well-being.

### 3.3. Construct Validity and Common Method Bias

We confirmed the discriminant and convergent validity of all latent constructs by using confirmatory factor analysis (CFA) in Mplus 7.0. First, we tested the measurement models separately and operated GR, NR, IM, POB, and strength use as the first-order constructs, respectively, and operated well-being as the second-order construct. Second, the composite reliability and average variance extracted (AVE) of all latent constructs exceeded the 0.70 [69] and 0.50 threshold [70], respectively, thus supporting the convergent validity. Third, the square root of AVE of all latent constructs is higher than each correlation with other constructs [70], and the results of CFA (Table 1) revealed that the six-factor model was a best-fitting model, providing the evidence of discriminant validity (χ^2^_(480)_ = 784.68, TLI = 0.97, SRMR = 0.03, CFI = 0.97, RMSEA = 0.03).

Furthermore, participants responded to the items from two different time points, which allows us to rule out the possibility of common method bias. The data fit of the six-factor model is better than that of the one-factor model (Table 1), which further shows that our research does not have serious method bias.

### 3.4. Analytical Strategy

We analyzed the data by using the structural equation model (SEM) in Mplus 7.0 because it used latent variables to explain measurement errors and tested the mediating model effectively [71]. Following Bandalos and Finney [72], we have seen well-being as a first-order latent variable by using the parceling technique in the SEM. We estimated the direct effects of GR and NR on employees’ well-being, and their mechanisms based on the framework (Figure 1). The results of SEM show the adequate data fit of the direct and mediating models (χ^2^_(113)_ = 174.95, TLI = 0.98, SRMR = 0.03, CFI = 0.98, RMSEA = 0.03; χ^2^_(263)_ = 401.24, TLI = 0.98, SRMR = 0.04, CFI = 0.98, RMSEA = 0.03). We examined the whole model (Figure 1) whereby GR and NR would be separately associated with employees’ well-being (i.e., H1a, H1b). We also tested whether GR and NR impact well-being and whether they influenced employees’ well-being via different mechanisms (i.e., H2a–3b) and how strength use moderates the effect of GR on AM and the effect of NR on POB (i.e., H4a, H4b).

### 3.5. Results

#### 3.5.1. Descriptive Findings

Table 2 showed Cronbach’s alpha values, standard deviations, and means of all variables. The correlations showed that GR was positively related to employees’ well-being (*p* < 0.01, r = 26), and NR was negatively related to well-being (*p* < 0.01, r = −0.29). GR was positively related to IM (*p* < 0.01, r = 16) and negatively related to POB (*p* < 0.01, r = −0.21), and IM was positively related to well-being (*p* < 0.01, r = 0.41). NR was positively related to POB (*p* < 0.01, r = 0.42) and negatively related to IM (*p* < 0.01, r = −0.21), and POB was negatively related to employees’ well-being (*p* < 0.01, r = −0.32). These relationships are expected, thus allowing us to further estimate the path links in the direct and mediating models.

#### 3.5.2. Hypotheses Testing

All the estimations of path links in our framework were controlled for age, gender, and tenure. The direct associations we examined were significant, which showed that GR positively impacts well-being (*p* < 0.01, β = 0.24) and NR negatively impacts well-being (*p* < 0.01, β = −0.29) (Table 3). H1a and H1b were supported. The results in Table 3 supported the mediating roles of IM and POB underlying the effects of GR and NR on well-being. The path links in the direct effect model remain significant (*p* < 0.01, β = 0.17; *p* < 0.05, β = −0.13). GR was positively related to IM (*p* < 0.05, β = 0.13) and negatively related to POB (*p* < 0.01, β = −0.13). NR was positively related to POB (*p* < 0.01, β = 0.43) and negatively related to IM (*p* < 0.01, β = −0.20). IM positively related to well-being (*p* < 0.01, β = 0.42), and POB was negatively related to well-being (*p* < 0.01, β = −0.19). Accordingly, H2a–3b about the mechanisms (i.e., IM, POB) underlying the effect of GR and NR on well-being were supported.

We ran a bootstrapping procedure (N = 5000) following Zhao, Lynch, and Chen [73] and Preacher and Hayes [74] to further test the indirect effect of GR and NR on employees’ well-being. The results of a 95% bias-corrected confidence interval confirmed that the indirect effect of GR via IM (0.01, 0.10) or POB (0.01, 0.05) was significant for employees’ well-being and that of NR via IM (−0.13, −0.04) or POB (−0.14, −0.03) was also significant for employees’ well-being.

Moreover, we conducted the Sobel z-test [71,73] to isolate the indirect effect of imbalanced reciprocity through corresponding mediators (i.e., IM, POB). In Table 4, the indirect effect of GR on well-being through IM is significant (*p* < 0.05, β = 0.06) and that through POB is significant (*p* < 0.01, β = 0.05). In contrast, the indirect effect of NR on well-being through POB is significant (*p* < 0.01, β = −0.08) and through IM is significant (*p* < 0.01, β = −0.07). Overall, these findings revealed that the four isolated mediating effects were confirmed, thus supporting H2a–H3b.

Using Sheng et al. [71] and Palmatier, Dant, and Grewal’s [75] method, we conducted multigroup structural analyses to test the moderating role of strength use on specific relationships. According to the median value (median value = 5) of strength use, we divided all samples into two groups (N_high_ = 241, N_low_ = 275). As shown in Table 5, we compared the difference in χ^2^ between the constrained model and unconstrained model and estimated the path links between GR and IM, and between NR and POB in the high and low groups, respectively. H4a was supported because the positive relationship between GR and IM became strong when strength uses was high (*p* < 0.01, β = 0.33, △χ^2^ (1) = 6.25, *p* < 0.05). H4b was supportive because the positive relationship between NR and POB becomes weak when strength uses is high (n.s., β = 0.14, △χ^2^ (1) = 17.79, *p* < 0.01).

## 4. Discussion

This study analyzes the differential effects of GR and NR on employees’ well-being at the workplace. There are several academic and practical contributions. First, based on SET [1], our research expands the literature on intrinsic rewards of employees to the organization by investigating the effects of GR and NR on employees’ well-being. Our research proves that an organization’s expectation of exchanging relationships with employees can affect the intrinsic rewards of employees to the organization by investigating the different impacts of exchange relationship norms on employees’ well-being. Regarding employees’ contribution to the organization, the majority of the literature on SET focuses on their extrinsic rewards (e.g., instrumental service), ignoring intrinsic rewards [1,11]. However, employees’ intrinsic rewards reflect their affection for the organization, which can boost their long-term relationship with the organization. Our research has demonstrated that GR is positively related to employees’ well-being, thus proving that an organization with GR can increase employees’ intrinsic rewards for the organization. Furthermore, this study indicated that NR is negatively associated with well-being, thus proving that an organization with NR can decrease employees’ intrinsic rewards for the organization. Therefore, practitioners can consider increasing the level of GR or reducing the level of NR to improve employees’ perception of the organization as willing to establish a long-term relationship with them and, ultimately, to provide intrinsic rewards to the organization (i.e., well-being).

Second, we researched the organizational antecedents of employees’ well-being from an imbalanced reciprocity perspective, expanding the literature on employees’ well-being. SET focus on the formation of social exchange relationships [1,52]. Employees’ perception of well-being in the workplace can help maintain a long-term exchange relationship with the organization. The existing literature on antecedents of well-being mainly includes organizational context, traits, psychological states [32,76], and work behavior [39,40]. However, imbalanced reciprocity (i.e., GR, NR) embedding the human resource management system influencing well-being has been hitherto ignored. Our findings demonstrated that GR and NR can differently influence employees’ well-being, further expanding the antecedents of well-being from an imbalanced reciprocity perspective. Therefore, practitioners can build GR or justify NR situations to improve employees’ well-being.

Third, based on SET [1], we bridged the unclear links between imbalanced reciprocity and well-being by uncovering the mediating roles of IM and POB, thus helping researchers better understand the psychological mechanism of social exchange. If the mechanisms of their indirect relationships are neglected, researchers and managers cannot understand the psychological process through which GR and NR decrease versus increase employees’ well-being and build up the efficient reciprocity context. The organization with GR tends to achieve employees’ interest, which may promote their trust in the organization. This trust helps employees easily perceive meaningful information provided by the organization for self-competence to increase employees’ IM and decrease POB, which then influences employees’ well-being. In contrast, the organization with NR tends to decrease employees’ trust in their organization, which then decreases IM and increases POB to influence employees’ well-being. Therefore, our contribution is to build a bridge that simultaneously aligns with imbalanced reciprocity (i.e., GR, NR) and well-being through the use of the IM and POB. Our findings add to the literature by demonstrating that IM and POB as psychological processes can explain why GR and NR are differentially related to work-related well-being, which stands to help managers and researchers understand how imbalanced reciprocity influences well-being to pay attention to the improvement of their mechanisms.

Fourth, by integrating the SET [1,4,18] and SDT [22], we examined whether the efficacy of GR and NR is contingent on employees’ strength use. The extant literature suggests that employees can utilize strengths to maximize the positive benefits rather than remedying the limitations [58,60]. However, few studies have hitherto explained the moderating role of strength use on the links between organizational context and employees’ psychological state. The results reveal that GR has an increased impact on IM when strength use is high rather than low, and NR has an increased impact on POB when strength use is high rather than low. Strength use tends to enhance the trust of the organization that implements GR to the employees, thereby increasing employees’ trust in their organization, and finally enhancing the increasing trend of employees’ IM. Meanwhile, strength use tends to weaken the NR organization’s distrust of employees to reduce employees’ distrust of the organization, and finally weaken the upwards trend of POB of employees. Practitioners can help employees use their strengths to amplify the positive effect of GR on employees’ intrinsic motivation and buffer the negative effect of NR on employees’ perceived organizational obstruction.

### Limitations and Future Research Directions

Our research is subject to limitations regarding the research design and sample. First, a cross-sectional design was conducted in this study to examine the whole model (Figure 1) and participants responded to all items through the self-reports, which cannot ignore the possibility of common methods bias. However, we distributed different parts of questions to participants at two time points, and the results (Table 1) revealed that the six-factor model has the best data fit during the measurement models, thus providing evidence to avoid the serious possibility of common method bias. Future research needs to simultaneously consider the manipulation of independent variables and moderators (i.e., strength use) before testing IM and POB in the experimental study, thus further diminishing the possibility of common method bias. Meanwhile, other individuals’ indifference (e.g., psychological capital) may moderate the effect of reciprocity on intrinsic motivation and perceived organizational obstruction. Practitioners may manage employees’ psychological capital to improve their psychological state [77].

Another limitation is that the data source is from China, which has seen a decreasing trend in the individual level of well-being. The 2019 World Happiness Report conducted by the United Nations revealed that the level of individuals’ well-being in China declined in comparison to the level in 2018. Although the degree of individuals’ well-being in China is generally low, the results in this study reveal that the differential effects of GR and NR on individuals’ well-being are significant. It is advisable to collect data from a different country in future research, which can underline the difference in the degree of an individual’s well-being. By doing so, we believe that the effects of imbalanced reciprocity on well-being will be more encouraging.

## 5. Conclusions

This study attempts to explore how and when generalized reciprocity and negative reciprocity (i.e., imbalanced reciprocity) influence employees’ well-being. In particular, the findings demonstrate that generalized reciprocity is positively related to employees’ well-being, whereas negative reciprocity is negatively related to their well-being. The results indicate that both intrinsic motivation and perceived organizational obstruction can mediate the relationship between generalized reciprocity and well-being and mediate the relationship between negative reciprocity and well-being as well. We highlight that strength use can enhance the positive effect of generalized reciprocity on intrinsic motivation and can weaken the positive effect of negative reciprocity on perceived organizational obstruction. Our research further expands the literature on reciprocity and well-being. Our research not only expands the antecedents of well-being from the imbalanced reciprocity perspective but also helps practitioners realize that imbalanced reciprocity can differently affect employees’ intrinsic rewards to organizations (i.e., well-being).

## Figures and Tables

**Table 1:** The comparison among all measurement models.

Model	χ^2^	df	χ^2^/df	△χ^2^ (△df)	SRMR	RMSEA	CFI	TLI
Six-factor model	784.68	480	1.63		0.03	0.03	0.97	0.97
Five-factor model	1439.01	485	2.97	654.33 ** (5)	0.07	0.06	0.91	0.90
Four-factor model	2024.20	489	4.14	1239.52 ** (9)	0.09	0.08	0.85	0.84
Three-factor model	2309.83	492	4.69	1525.15 ** (12)	0.10	0.08	0.83	0.81
Two-factor model	3922.57	494	7.94	3137.89 ** (14)	0.12	0.11	0.67	0.65
One-factor model	6526.63	495	13.19	5741.95 ** (15)	0.17	0.15	0.42	0.38

** *p* < 0.01, N = 584. Six-factor model: Six latent constructs; Five-factor model: GR and NR were combined into one factor; Four-factor model: GR and NR were combined into one factor, and IM and POB were combined into one factor; Three-factor model: GR and NR were combined into one factor, IM and POB were combined into one factor, and strength use and well-being were combined into one factor; Two-factor model: GR, NR, IM, and POB were combined into one factor, and strength use and well-being were combined into one factor; One-factor model: all variables were combined into one factor.

**Table 3:** The direct and indirect effects of structural equation model.

	Direct Effect Model	Mediating Effect Model		
	Well-Being	Intrinsic Motivation	Perceived Organizational Obstruction	Well-Being
Generalized reciprocity	0.24 ** (0.05)	0.13 * (0.04)	−0.13 ** (0.06)	0.17 ** (0.04)
Negative reciprocity	−0.29 ** (0.05)	−0.20 ** (0.04)	0.43 ** (0.06)	−0.13 * (0.04)
Intrinsic motivation				0.42 ** (0.06)
Perceived organizational obstruction				−0.19 ** (0.03)
Age	0.00 (0.06)			0.00 (0.01)
Gender	0.03 (0.05)			0.03 (0.07)
tenure	0.07 (0.06)			0.08 (0.01)
X^2^	174.95	401.24		
df	113	263		
CFI	0.98	0.98		
TLI	0.98	0.98		
RMSEA	0.03	0.03		
SRMR	0.03	0.04		

* *p* < 0.05. ** *p* < 0.01. Path estimates are standardized. The values in the parentheses are standard errors.

**Table 2:** Descriptive statistics.

	Mean	SD	Gender	Age	Tenure	GR	NR	IM	POB	STR	WEB
Gender	1.37	0.49	1								
Age	30.58	5.71	0.01	1							
Tenure	3.66	3.89	−0.04	0.69 **	1						
GR	4.85	0.98	0.01	−0.03	−0.03	(0.80)					
NR	3.38	1.26	−0.08	0.02	0.01	−0.21 **	(0.91)				
IM	4.82	1.09	0.00	−0.02	−0.02	0.16 **	−0.21 **	(0.80)			
POB	3.11	1.40	0.00	−0.01	−0.02	−0.21 **	0.42 **	−0.22 **	(0.93)		
STR	4.77	1.05	−0.02	0.04	0.05	0.18 **	−0.19 **	0.25 **	−0.18 **	(0.94)	
WEB	5.01	0.81	0.03	0.04	0.06	0.26 **	−0.29 **	0.41 **	−0.32 **	0.41 **	(0.92)

** *p* < 0.01, Cronbach’s alpha values were in parentheses.

**Table 4:** The results of the Sobel Z-test.

Indirect Path		Unstandardized Path		Isolated Mediating Effect	Sobel z-Test	*p*-Value
		Coefficient				
		a	b	(a × b)		
H2a	GR→IM→Well-being	0.13	0.46	0.06	2.57	*p* < 0.05
H2b	NR→IM→Well-being	−0.15	0.45	−0.07	−3.50	*p* < 0.01
H3a	GR→POB→Well-being	−0.27	−0.19	0.05	3.53	*p* < 0.01
H3b	NR→POB→Well-being	0.49	−0.16	−0.08	−3.77	*p* < 0.01

**Table 5:** The results of moderating effects.

	Strength Use		
Paths	β of High Group	β of Low Group	△X^2^ (△df = 1)
GR→IM	0.33 ** (0.07)	0.07 (0.05)	6.25 *
NR→POB	0.14 (0.07)	0.50 ** (0.07)	17.79 **

* *p* < 0.05. ** *p* < 0.01.

## Data Availability

The data are available from the corresponding author on reasonable request.

## Appendix A

**The measurement items of the study:**

Generalized Reciprocity (GR):

1. My organization would help me develop myself, even if I cannot make more contributions at present.

2. My organization seems willing to invest in my professional development, even when it does not directly impact my current job performance.

3. My organization would do something for me without any strings attached.

4. My organization takes care of me in ways that exceed my contribution to the organization.

Negative Reciprocity (NR):

1. I have the impression that my organization is up to something that could hurt me.

2. My organization would never help me out unless it was in the organization’s own interest.

3. What I have received from my organization is only a small part of my contribution to the organization.

4. My organization expects more from me than it gives me in return.

5. My organization only cares about its own benefits and never cares about my career or living.

6. If my organization gives me double wages, it will require me to put in three or four times more energy.

7. My organization seems to think that I need to work hard no matter how poorly I am treated.

Strength Use:

1. I am regularly able to do what I do best.

2. I always try to use my strengths.

3. I achieve what I want by using my strengths.

4. I use my strengths every day.

5. I use my strengths to get what I want out of life.

6. My life presents me with lots of different ways to use my strengths.

7. Using my strengths comes naturally to me.

8. I find it easy to use my strengths in the things I do.

9. I am able to use my strengths in lots of different situations.

10. Using my strengths is something I am familiar with.

11. I am able to use my strengths in lots of different ways.

Intrinsic Motivation (IM):

1. Because I have fun doing my job.

2. Because what I do in my work is exciting.

3. Because the work I do is interesting.

Perceived Organizational Obstruction (POB):

1. My organization obstructs the realization of my professional goals.

2. My organization is a detriment to my well-being.

3. The organization gets in the way of my performance.

4. The company blocks my personal goals.

5. My goal attainment is thwarted by the organization.

Well-being:

1. I feel satisfied with my life.

2. I am close to my dream in most aspects of my life.

3. Most of the time, I do feel real happiness.

4. I am in a good life situation.

5. My life is very fun.

6. I would hardly change my current way of life in the afterlife.

7. I am satisfied with my work responsibilities.

8. In general, I feel fairly satisfied with my present job.

9. I find real enjoyment in my work.

10. I can always find ways to enrich my work.

11. Work is a meaningful experience for me.

12. I feel basically satisfied with my work achievements in my current job.

13. I feel I have grown as a person.

14. I handle daily affairs well.

15. I generally feel good about myself, and I am confident.

16. People think I am willing to give and to share my time with others.

17. I am good at making flexible timetables for my work.

18. I love having deep conversations with family and friends so that we can better understand each other.
